# High‐throughput lipidomic profiles sampled with electroporation‐based biopsy differentiate healthy skin, cutaneous squamous cell carcinoma, and basal cell carcinoma

**DOI:** 10.1111/srt.13706

**Published:** 2024-05-09

**Authors:** Leetal Louie, Julia Wise, Ariel Berl, Ofir Shir‐az, Vladimir Kravtsov, Zohar Yakhini, Avshalom Shalom, Alexander Golberg, Edward Vitkin

**Affiliations:** ^1^ Porter School of Environment and Earth Sciences Tel Aviv University Tel Aviv Israel; ^2^ Department of Plastic Surgery Meir Medical Center Kfar Sava Israel; ^3^ Department of Pathology Meir Medical Center Kfar Sava Israel; ^4^ Arazi School of Computer Science Reichman University Herzliya Israel; ^5^ Department of Computer Science Technion ‐ Israel Institute of Technology Haifa Israel

**Keywords:** basal cell carcinoma, cutaneous squamous cell carcinoma, e‐biopsy, electroporation‐based biopsy, high‐throughput lipidomics, lipidomic profiles

## Abstract

**Background:**

The incidence rates of cutaneous squamous cell carcinoma (cSCC) and basal cell carcinoma (BCC) skin cancers are rising, while the current diagnostic process is time‐consuming. We describe the development of *a novel approach to high‐throughput sampling of tissue lipids using electroporation‐based biopsy, termed e‐biopsy. We report on the ability of the* e‐biopsy technique to harvest large amounts of lipids from human skin samples.

**Materials and Methods:**

Here, 168 lipids were reliably identified from 12 patients providing a total of 13 samples. The extracted lipids were profiled with ultra‐performance liquid chromatography and tandem mass spectrometry (UPLC‐MS‐MS) providing cSCC, BCC, and healthy skin lipidomic profiles.

**Results:**

Comparative analysis identified 27 differentially expressed lipids (*p* < 0.05). The general profile trend is low diglycerides in both cSCC and BCC, high phospholipids in BCC, and high lyso‐phospholipids in cSCC compared to healthy skin tissue samples.

**Conclusion:**

The results contribute to the growing body of knowledge that can potentially lead to novel insights into these skin cancers and demonstrate the potential of the e‐biopsy technique for the analysis of lipidomic profiles of human skin tissues.

**Abbreviations**
BCC: basal cell carcinoma
Cer: ceramides
CerG2: diglycosylceramide
cSCC: cutaneous squamous cell carcinoma
DG: diglyceride
e‐biopsy: electroporation‐based biopsy
LPC: lyso‐phosphatidylcholine
LPE: lyso‐phosphatidylethanolamine
LPI: lyso‐phosphatidylinositol
PC: phosphatidylcholine
PI: phosphatidylinositol
PS: phosphatidylserine
TG: triglyceride
UPLC‐MS‐MS: ultra performance liquid chromatography and tandem mass spectrometry

## 1 INTRODUCTION

Rising trends in keratinocyte carcinoma (cSCC and BCC) incidence have been observed.^1^, ^2^, ^3^, ^4^, ^5^ These are among the most common cancers diagnosed, but due to their low mortality rate, they are usually excluded from cancer registries,^4^ despite their impact on quality of life and risks of premature mortality.^2^, ^6^ These trends foreshadow increasing wait time for diagnosis under the current gold standard diagnostic method which relies on excision and histopathological examination, with advanced technologies acting as support.^7^ The current gold standard is problematic when subsequent treatment requires electrodesiccation and cautery,^8^ so finding less invasive methods for differentiating between healthy and cancerous skin, as well as between cancer types will prove useful in light of incidence trends. Methods that can provide rapid results will also be beneficial considering the difference in aggressiveness and likelihood of metastasis of cSCC and BCC.^9^

Collecting samples of molecular information from cSCC and BCC lesioned skin with modern technology like e‐biopsy and others^10^, ^11^ is simpler and faster than current biopsy methods, alluding to the potential of molecular profiling in diagnostics. cSCC and BCC are cancers of keratinocytes that functionally make and secrete lipids,^12^, therefore, exploring the differential expression of their lipid profiles may reveal trends that differentiate the two from each other as well as from healthy skin.

Molecular profiling technology gives us snapshots of the cellular fabric where we can observe trends that have physical manifestations. We have entered the era of personalized medicine where biological profiling provides useful information in cancer research and treatment.^13^, ^14^, ^15^ Previously, high‐throughput analyses of genes, proteins, and metabolites have been reported for cSCC and BCC.^16^, ^17^, ^18^, ^19^, ^20^ Lipids profiles, however, have mostly been reported for BCC^21^, ^22^, ^23^ using low‐throughput methods and serum samples with the exception of one high‐throughput cSCC study that included 70 lipids among metabolites.^24^ Beyond medicine, molecular profiling plays an important role in other aspects of life such use in agriculture food and safety, forensic science, and environmental monitoring.

The recent electroporation‐based biopsy sampling technique, e‐biopsy, that leverages cell permeabilization caused by electric fields for molecular harvesting, demonstrated its ability to sample molecular profiles that differentiate between cancerous and healthy skin.^25^, ^26^, ^27^ Coupling this sampling method with UPLC‐MS‐MS, which has proven its ability to identify potential markers of skin cancer,^18^ provides a promising direction for high‐throughput analysis of extractable molecules.

Here we perform a comparative analysis of the first high‐throughput lipidomic profiling of cSCC, BCC, and healthy skin tissue using e‐biopsy. These profiles were collected with the e‐biopsy approach, thus adding to previous e‐biopsy enabled profiling in transcriptomics, proteomics, and metabolomics.^16^, ^25^, ^26^, ^27^

## 2 MATERIAL AND METHODS

### 2.1 Human patients

A list of patient age, sex, and tumor type is provided in **Table** **1**. This study was approved by the Meir Medical Center IRB, number MMC‐19‐0230. All patients gave consent for participation and for the performance of genetic analysis of their sample tissue.

**TABLE 1:** Patient sex, age, and tumor type.

Patient	Sex	Age	Tumor type
1	Male	73	cSCC
2	Female	85	cSCC
3	Male	56	cSCC
4	Male	85	cSCC
5	Female	69	BCC
6	Male	71	BCC
7	Female	91	BCC
8	Female	74	BCC
9	Male	81	BCC
10	Female	91	BCC
11	Female	74	Healthy
12	Female	57	Healthy

### 2.2 Sample collection

**FIGURE 1:**
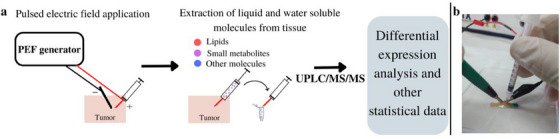
(A, B) E‐biopsy analysis and workflow. (A) Sample collection through pulsed electric field (PEF) application leads to the extraction of water‐soluble compounds that underwent subsequent UPLC‐MS‐MS and differential expression analysis. (B) Needle and electrode positioning on the skin during molecular harvesting by e‐biopsy.

From March 2020 to March 2022, 10 tissue samples were collected from 10 patients who underwent surgical excision of a skin lesion suspected as BCC or cSCC at Meir Medical Center, Israel. Three healthy tissue samples were collected from two patients undergoing blepharoplasty. Excised samples were at least 1 cm in diameter. E‐biopsy extraction was performed on 13 fresh (between 10–20 min after surgery) samples. Lipid analysis was performed via UPLC‐MS‐MS. **Figure** **1** summarizes the workflow and e‐biopsy method.

E‐biopsy was performed with a custom‐made high‐voltage pulsed electric field generator under conditions previously described for protein extraction from cSCC and BCC.^16^ The liquids sampled were immediately transferred to 1.5 mL tubes with 100 µL double distilled water and stored at −20°C until shipped to Beijing Genomics Institute for analysis.

### 2.3 UPLC‐MS‐MS analysis

The UPLC‐MS‐MS analysis was performed by the Beijing Genomics Institute. An ACQUITY UPLC CSH C18(1.7 µm, 2.1*100 mm, Waters, USA) and Q Exactive mass spectrometer (Thermo Fisher Scientific, USA) were used for lipid analysis. The output of the UPLC‐MS/MS analysis was imported to LipidSearch v.4.1 software (Thermo Fisher Scientific, USA) for molecular identification and quantification. The software was also used to impute missing values. Excel files were generated to include, among other things, the lipid ID, reliability score (graded A to D, with A and B being the most accurately identified lipids used for subsequent differential lipid screening), and the observed intensity of the lipid in the sample (Table S1 and **GitHub**: https://github.com/GolbergLab/BCC_SCC_Lipidomics). This data was used for analyses of differential lipid abundance. Detailed UPLC‐MS/MS information and methods can be found in the Supplementary Methods.

### 2.4 Differential lipid screening

**FIGURE 2:**
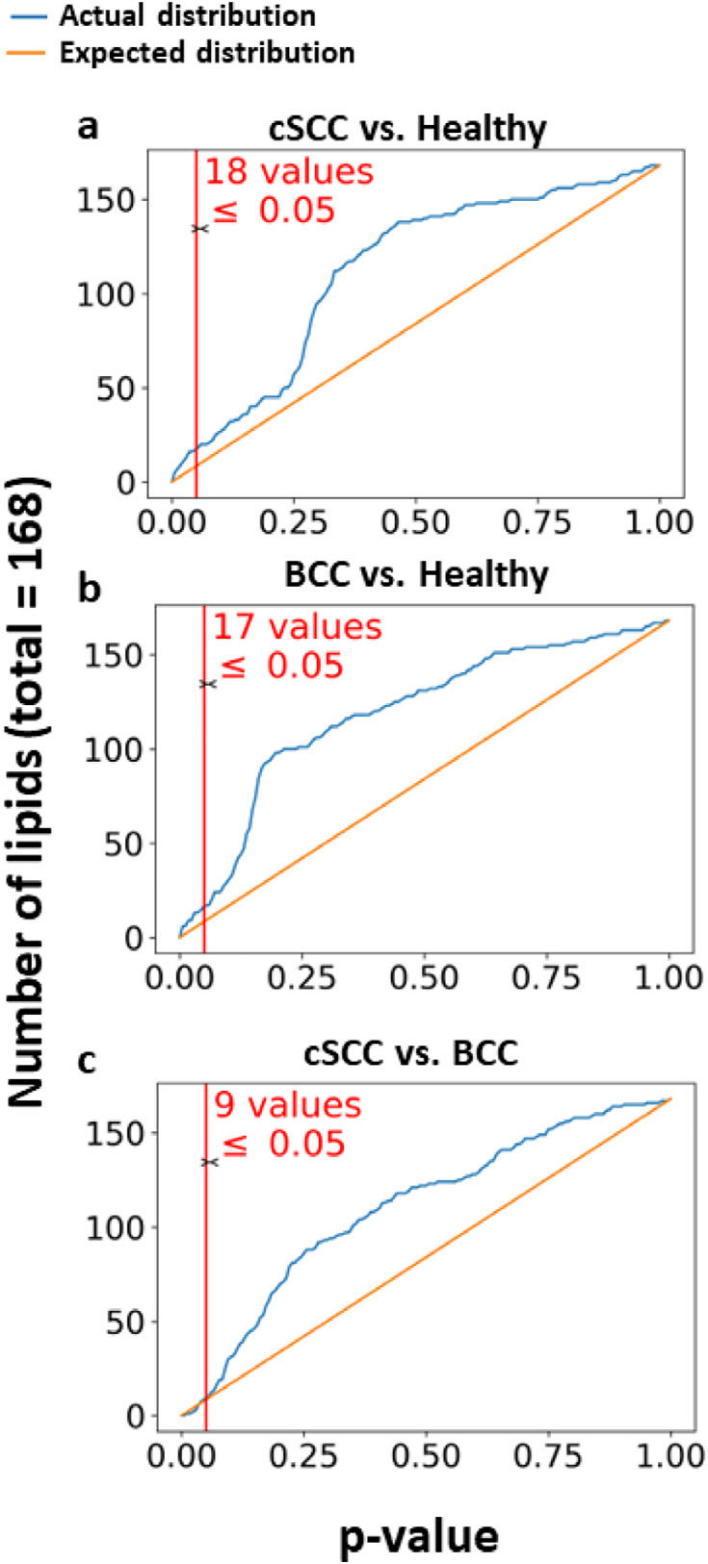
(A–C) Overabundance plots comparing the distribution of lipid differential expression (both over‐ and under‐expression) *p*‐values between control (normal skin tissue), BCC, and cSCC tumor samples. A total of 13 samples and 168 lipids extracted by e‐biopsy were analyzed. The vertical red line marks the Student's *T*‐test *p*‐value cut‐off of 0.05. (A) cSCC versus Healthy, (B) BCC versus Healthy, and (C) cSCC versus BCC.

**FIGURE 3:**
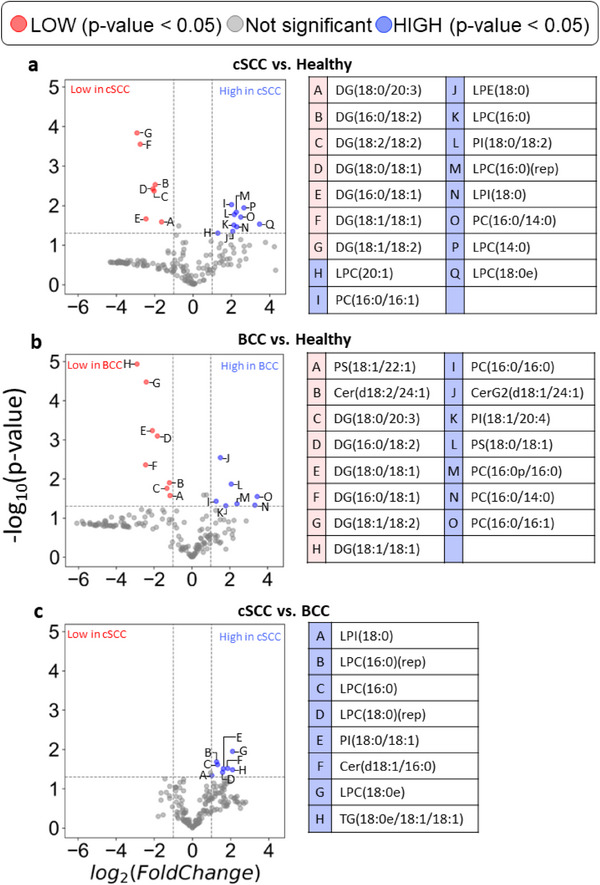
(A–C) Volcano plots and tables showing the fold change difference of lipid intensities. (A) cSCC vs. Healthy. (B) BCC versus Healthy. (C) cSCC versus BCC. Tables with lipid names correspond to lettered data points in the adjacent volcano plots. Fold change and *p*‐value data for lipids listed in the tables can be found in Table 2, Tables S2–S4.

The measured lipid intensities (Table S1) were used for differential lipid screening. Student's *T*‐test, and fold change between average measured lipid intensities were calculated for each comparison pair (cSCC vs. Healthy, BCC vs. Healthy, cSCC vs. BCC). The resulting *p*‐values and fold change values were used in overabundance analysis and to generate a volcano plot (Figures 2 and 3).

#### 2.4.1 Statistical overabundance analysis

Overabundance analysis compares actual and expected distributions of *p*‐values to verify that compounds have different abundance levels when compared to classes of samples.^28^ This approach explores internal data variability and helps address multiple comparisons. The analysis relies only on the number of compounds (i.e., lipids) and their observed corresponding *p*‐values (here obtained from the Student's *T*‐test). The distribution of the expected *p*‐values was generated from a null model assuming the same number of compounds (Figure 2).

#### 2.4.2 Volcano plot analysis

Volcano plot overlays the magnitude of the fold change of lipids with their differential significance between two analyzed populations. Differentially expressed lipids were defined for this analysis as those with a *‐log10(p‐value) >* *1.3* (i.e., *p‐value <* *0.05*) and those with *−1 <* *log2(fold‐change) <* *1*. Fold change calculations were carried out using the average of intensity values for each comparison group, that is, *fold‐change(lipid) = avg(grp1) / avg(grp2)*. The data was then filtered to include only the compounds with high‐reliability scores (grades A and B) (Figure 3). The data of the most interesting compounds were compiled into tables to showcase their associated *p*‐values and fold change values (**Table** **2**).

**TABLE 2:** Differentially expressed lipids with associated *p*‐values and fold change values.

	cSCC vs. Healthy	BCC vs. Healthy	cSCC^SH^ and BCC^BH^ vs. Healthy	cSCC vs. BCC	cSCC vs. BCC ^SB^ and Healthy^SH^
	**DG(18:2/18:2)**	**Cer(d18:2/24:1)**	**DG(16:0/18:1)**	**Cer(d18:1/16:0)**	**LPC(16:0)**
*p*‐value	0.004	0.01	0.02**^SH^**, 0.004**^BH^**	0.03	0.02**^SB^** 0.03**^SH^**
Fold change	0.25	0.44	0.18**^SH^**, 0.18**^BH^**	3.59	2.4883**^SB^**, 4.4**^SH^**
	**LPC(14:0)**	**CerG2(d18:1/24:1)**	**DG(16:0/18:2)**	**LPC(18:0)(rep)**	**LPC(16:0)(rep)**
*p*‐value	0.01	0.003	0.003**^SH^**, 0.0008**^BH^**	0.04	0.02**^SB^**, 0.01**^SH^**
Fold change	6.3	2.84	0.26**^SH^**, 0.28**^BH^**	2.98	2.41**^SB^**, 4.77**^SH^**
	**LPC(20:1)**	**PC(16:0/16:0)**	**DG(18:0/18:1)**	**PI(18:0/18:1)**	**LPC(18:0e)**
*p*‐value	0.05	0.04	0.004**^SH^**, 0.0006**^BH^**	0.03	0.01**^SB^**, 0.03**^SH^**
Fold change	2.45	2.43	0.24**^†^**, 0.24**^BH^**	3.07	4.29**^SB^**, 11.07**^SH^**
	**LPE(18:0)**	**PC(16:0p/16:0)**	**DG(18:0/20:3)**	**TG(18:0e/18:1/18:1)**	**LPI(18:0)**
*p*‐value	0.05	0.04	0.03**^SH^**, 0.02**^BH^**	0.03	0.05**^SB^**, 0.03**^SH^**
Fold change	4.21	5.21	0.32**^SH^**, 0.40**^BH^**	4.3	2.03**^SB^**, 4.86**^SH^**
	**PI(18:0/18:2)**	**PI(18:1/20:4)**	**DG(18:1/18:1)**		
*p*‐value	0.02	0.05	0.0003**^SH^**, 1.16E‐05**^BH^**		
Fold change	4.46	3.46	0.15**^SH^**, 0.13**^BH^**		
		**PS(18:0/18:1)**	**DG(18:1/18:2)**		
*p*‐value		0.01	0.0001**^SH^**, 3.33E‐05**^BH^**		
Fold change		4.22	0.13**^SH^**, 0.19**^BH^**		
		**PS(18:1/22:1)**	**PC(16:0/14:0)**		
*p*‐value		0.03	0.02**^SH^**, 0.05**^BH^**		
Fold change		0.45	5.61**^SH^**, 10.02**^BH^**		
Summary of lipids	Diglyceride	Ceramides	Diglyceride	Ceramides	Lyso‐phospholipids
	Lyso‐phospholipids	Phospholipids	Phospholipid	Lyso‐phospholipids	
	Phospholipid			Phospholipids	
				Triglycerides	
DG Diglyceride			LPI Lyso‐phosphatidylinositol		
Cer Ceramides			PI Phosphatidylinositol		
CerG2 Diglycosylceramide			PC Phosphatidylcholine		
LPC Lyso‐phosphatidylcholine			PS Phosphatidylserine		
LPE Lyso‐phosphatidylethanolamine			TG Triglyceride		

*Note*: Columns contain the lipids (in bold) identified as significant by the pairwise comparison of groups: CSCC vs. Healthy, BCC vs. Healthy, cSCC vs. BCC. Also categorized are the lipids found in combined cSCC and BCC groups compared to Healthy, showing the difference between keratinocyte carcinoma and healthy skin. Last, lipids of cSCC are isolated with results of common lipids that differentiate cSCC from BCC and healthy skin. Reading example: Cer(d18:2/24:1) has Student's *T*‐test *p*‐value of 0.01 and the ratio of its average intensity in BCC tissues to its average intensity in healthy tissues is 0.44.

## 3 RESULTS

The initial 309 identified lipids were filtered according to reliability score, omitting lipids with grades C and D, which resulted in 168 lipids eligible for differential expression analysis. The analysis was performed for each of three comparison configurations: cSCC versus Healthy, BCC versus Healthy, and cSCC versus BCC. Overabundance plots (Figure 2) represent analysis results, where the number of lipids with Student's *T*‐test *p*‐value below 0.05 is highlighted in red. Volcano plots (Figure 3) show relative group affinity of each lipid, highlighting significantly over‐ and under‐expressed lipids. The major findings are summarized in Table 2.

### 3.1 Electroporation sampled lipidomic profile differentiate cSCC versus Healthy skin

The overabundance analysis of cSCC compared to Healthy showed a total of 18 lipids with Student's *T*‐test *p*‐values below 0.05 (Figure 2A, corresponding to FDR = 0.47). Moreover, six of these lipids resulted in *p*‐value below 1e‐2 (FDR = 0.28) and two of them in *p*‐value below 1e‐3 (FDR = 0.08). Of these, seven were significantly lower in cSCC and 10 significantly higher in cSCC (Figure 3A). All observed under‐expressed cSCC lipids were diglycerides, specifically (in increasing negative fold change): DG(18:0/20:3), DG(16:0/18:2), DG(18:2/18:2), DG(18:0/18:1), DG(16:0/18:1), DG(18:1/18:1), and DG(18:1/18:2) (Figure 3A). Lipids with higher expression in cSCC were phospholipids and lyso‐phospholipids, specifically (in increasing positive fold change): LPC(20:1), PC(16:0/16:1), LPE(18:0), LPC(16:0), PI(18:0/18:2), LPC(16:0)(rep), LPI(18:0), PC(16:0/14:0), LPC(14:0), and LPC(18:0e) (Figure 3A). The high‐resolution volcano plot of this comparison can be viewed in **Figure** **S1**. All associated *p*‐values and fold change values for the lipids listed are reported in **Table** **S2**.

### 3.2 Electroporation sampled lipidomic profile differentiate BCC vs. Healthy skin

The overabundance analysis of BCC compared to Healthy showed a total of 17 lipids with Student's *T*‐test *p*‐values less than 0.05 (Figure 2B, corresponding to FDR = 0.49). Moreover, six of these lipids resulted in *p*‐value below 1e‐2 (FDR = 0.28) and four of them in *p*‐value below 1e‐3 (FDR = 0.04). Of these, eight were significantly lower in BCC and seven significantly higher in BCC (Figure 3B). Observed under‐expressed BCC lipids were mostly diglycerides as well as a ceramide and phospholipid, specifically (in increasing negative fold change): PS(18:1/22:1), Cer(d18:2/24:1), DG(18:0/20:3), DG(16:0/18:2), DG(18:0/18:1), DG(18:1/18:2), DG(16:0/18:1), and DG(18:1/18:1) (Figure 3B). Lipids with higher expression in BCC were mostly phospholipids and a ceramide, specifically (in increasing positive fold change): PC(16:0/16:0), CerG2(d18:1/24:1), PI(18:1/20:4), PS(18:0/18:1), PC(16:0p/16:0), PC(16:0/14:0), and PC(16:0/16:1) (Figure 3B). Higher levels of phospholipids in BCC versus healthy skin are consistent with previous reports.^22^ In contrast to previous reports that found significantly higher TGs in BCC compared to healthy skin,^22^ all the 65 TGs identified by this study were (not‐significantly) lower in BCC compared to healthy tissues. The high‐resolution volcano plot of this comparison can be viewed in Figure S2. All associated *p*‐values and fold change values for the lipids listed are reported in Table S3.

### 3.3 Electroporation sampled lipidomic profile differentiate cSCC versus BCC skin

The overabundance analysis of cSCC compared to BCC showed a total of nine lipids with Student's *T*‐test *p*‐values less than 0.05 (Figure 2C, corresponding to FDR = 0.93), with eight of them significantly higher in cSCC and none significantly lower (Figure 3C). Lipids with higher expression in cSCC compared to BCC were mostly lyso‐phospholipids as well as a phospholipid, ceramide, and triglyceride, specifically (in increasing order of fold change): LPI(18:0), LPC(16:0)(rep), LPC(16:0), LPC(18:0)(rep), PI(18:0/18:1), Cer(d18:1/16:0), LPC(18:0e), and TG(18:0e/18:1/18:1) (Figure 3C). The high‐resolution volcano plot of this comparison can be viewed in Figure S3. All associated *p*‐values and fold change values for the lipids listed are reported in Table S4.

## 4 DISCUSSION

We performed a comparison of high‐throughput lipidomic profiles sampled with e‐biopsy from healthy, cSCC, and BCC skin tissues. The overabundance and volcano plot analyses suggest a difference in lipidomic profiles between cancer and healthy skins, with a general trend of lower DGs and higher phospholipid subclasses in cancerous tissue. There was also a slight difference and a separation potential between cSCC and BCC lipid profiles as higher intensities of phospholipids and other lipids were observed in cSCC. The comparison of cSCC to healthy tissue revealed lower DGs and higher phospholipids and lyso‐phospholipids. Similarly, the comparison of BCC to healthy skin found lower diglycerides and higher phospholipids. In the BCC to healthy tissue comparison, two ceramides were identified, one higher and the other lower in BCC. In the comparison of cSCC to BCC, several lyso‐phospholipids, and a single phospholipid, ceramide, and triglyceride were identified at higher intensities in cSCC.

To the best of our knowledge, no previous molecular profiling study focused only on the lipidomic profiling of cSCC. Previous studies comparing BCC and healthy (with sample sizes of 30 and 64)^21^, ^23^ and a study comparing 12 BCC, 13 AK, and 11 healthy skin samples,^22^ reported lipidomic profiles for only six lipid groups: cholesterol, HDL, LDL, triglycerides, phospholipids, and total lipids. Triglycerides were previously reported as significantly higher in BCC versus Healthy skin samples,^22^ but not significant in serum samples for the same comparison.^21^, ^22^, ^23^ Phospholipids were previously found significantly higher in BCC versus Healthy in both skin and serum samples.^22^

Our study significantly improves on the level of detail of reported lipids of BCC and healthy skin. E‐biopsy coupled with UPLC‐MS‐MS was able to measure specific ceramides, lyso‐phospholipids, and diglycerides in addition to triglycerides and phospholipids. In contrast to a previous report,^22^ triglycerides were not identified as significant in BCC compared to Healthy skin samples. Rather, they were found in significantly lower levels in BCC compared to cSCC. Like in the previous study,^22^ our results show higher phospholipid levels in BCC compared to Healthy skin. Six phospholipid subclasses were expressed higher in BCC compared to Healthy, and one phospholipid type was lower in BCC compared to Healthy (Figure 3B).

Our study contributes novel information on the lipid profile of cSCC and its comparative analysis to BCC and to healthy skin. Diglycerides, triglycerides, ceramides, phospholipids, and lyso‐phospholipids were identified in cSCC. Diglycerides were expressed significantly lower and phospholipids and lyso‐phospholipids were significantly higher in cSCC compared to healthy skin (Figure 3A). In cSCC compared to BCC, phospholipids, lyso‐phospholipids, ceramides, and triglycerides were expressed significantly higher (Figure 3C). This study differs from previous cSCC studies in the methods of sample collection and lipid analysis. Previously, cSCC and healthy skin were sampled from the same patient with healthy skin taken from beyond tumor margins, and lipids were analyzed among metabolites from whole tissue samples.^24^ This study, in contrast, collected healthy skin tissue from a separate set of patients and performed an exclusive lipid analysis on the collected tissues. This resulted in a larger number of lipids detected and analyzed and better reflects the real‐life diversity of the human lipidome. Additionally, the whole tissue samples in previous studies were collected over relatively large areas, thus allowing the inherent tissue and tumor molecular heterogeneity to obscure the signals of interest. This is in contrast to localized sampling using e‐biopsy which better reflects the actual spatial molecular state in the condition of interest.

The lipids included in the molecular profiling, have variable reported effects on carcinogenesis. Ceramides (Cer) are potent tumor suppressor lipids as they can enhance keratinocytes apoptosis and also block cell cycle transition limiting cancer cell proliferation.^29^ Diglycosylceramides (CerG2) are involved in forming large amounts of lipids in the cells of the innate immune system.^29^ The higher Cer levels in cSCC compared to BCC may be a reflection of the difference in aggressiveness and the need for stronger tumor suppression in cSCC. Higher CerG2 levels in BCC compared to healthy suggest an immune response is occurring in the tumor area. Phosphatidylserine (PS) is translocated from the inner to outer endothelial cell's membrane when exposed to oxidative stress, making it a potential marker for apoptotic and tumor cells.^30^ Phosphatidylcholine (PC) is involved in tumor microenvironment cellular communication and interestingly it was demonstrated that cancer cells accumulate PC precursors or products, compared to non‐malignant counterparts.^31^ Similarly, increased triglycerides (TG) levels were seen in both actinic keratosis and BCC compared to normal skin cells.^22^

Furthermore, when diglyceride (DG or diacylglycerol), a second messenger lipid, is oxidized by UVA or UVB it may act as an endogenous tumor promoter by activation of protein kinase C (PKC) and NADPH oxidase in human neutrophils.^32^, ^33^, ^34^, ^35^ Lyso‐phosphatidylcholine (LPC) is mainly derived from the turnover of phosphatidylcholine (PC) in circulation by phospholipase A2 (PLA2).^36^ LPC can also induce the activation of PKC as well as phospholipase and regulate MAP kinase.^36^ LPC can recruit phagocytes to the site of apoptosis, hence plays an important role in the invasion, metastasis, and prognosis of tumors.^36^ This aligns with our findings of higher PC and LPC in cancer groups compared to healthy skin samples. Additionally, LPC was higher in cSCC compared to BCC, potentially reflecting the more aggressive nature of cSCC. Lyso‐phosphatidylinositol (LPI) is generated by PLA2 and a G protein receptor 55 (GPR55), upregulated in human cSCC and suggested to promote skin carcinogenesis and tumor aggressiveness.^37^ Phosphatidylinositol (PI) derivatives are synthesized in the phosphoinositide 3‐kinase (PI3K)/AKT pathway, which is one of the most frequently activated signaling pathways in human cancer, as well as been reported to be activated in both cSCC and BCC.^38^, ^39^, ^40^ This supports the observed increase if PI in all comparison groups of this study (cSCC vs. healthy, BCC vs. healthy, and cSCC vs. BCC). Last, Lyso‐phosphatidylethanolamine (LPE)’s physiological significance in the plasma remains unknown,^30^ however, it has been observed to significantly increase cell proliferation in breast cancer cell lines.^41^

There are a few limitations in this study to be noted. First, the sample size was not representative enough to draw a confident conclusion on the specific lipid behavior. Previous studies demonstrate the need for larger sample numbers to observe trends in skin cancer lipids,^21^, ^23^ thus an increased sample size can improve differential expression analysis, increase the confidence in findings, and reduce the amount of falsely detected signals. Second, the study was performed on the ex‐vivo samples, in‐vivo sampling of skin may provide slightly different results. Third, the inclusion of patients in the study was not very strict, and patient lifestyle information that could provide useful information, such as levels of sun exposure, was not collected.

The e‐biopsy sampling technique is still in its early stages but has the potential to be implemented in a handheld device, offering a promising solution to reduce the need for resection during biopsy. Unlike existing handheld devices like the iKnife^42^, ^43^ and MasSpec Pen,^44^, ^45^ which require a real‐time connection to a mass spectrometer and are primarily used intraoperatively, the e‐biopsy technique shows promise for broader applications. Other needle biopsy methods such as fine‐needle aspiration and core needle biopsy also eliminate the need for resection but are limited by needle size,^46^, ^47^ and the need for increasing diagnostic accuracy requires more invasive procedures with larger needle diameters.^48^, ^49^ In contrast, the e‐biopsy technique has demonstrated the ability to sample areas larger than an actual needle diameter,^27^ providing valuable site‐specific information. This also stands in contrast to previous lipid analysis studies that relied on serum samples, lacking a specific spatial context. This site‐specific sampling feature is advantageous as it enables the mapping of tumor heterogeneity and offers a deeper understanding of tumor complexities.^26^

## 5 CONCLUSION

This study significantly contributes to the understanding of cSCC and BCC diagnostics. It provides the first of its kind report of cSCC high throughput lipidomic profiles. It also provides high throughput lipidomic profiles for BCC and healthy skin samples, together with comparative analysis between all three tissues. Moreover, all lipidomic profiles reported here are of greater resolution compared to previous keratinocyte carcinoma lipid profiling. A total of 168 lipids were identified, of which 27 were recognized as differentially expressed in at least one comparison group. The differently expressed lipids are a variety of diglycerides, triglycerides, ceramides, phospholipids, and lyso‐phospholipids. Overall trends indicate lower diglycerides and higher phospholipids and lyso‐phospholipids in cSCC and BCC compared to Healthy skin tissue. In summary, this study significantly advances our understanding of cSCC and BCC diagnostics through high‐throughput lipidomic profiling. The identification of differentially expressed lipids and the comparative analysis among the three tissue types provide crucial insights into the lipidomic alterations associated with these skin cancers. The availability of the data online and the potential of the e‐biopsy technique pave the way for improved diagnostic approaches and hold promise for the future of skin cancer diagnosis.

## CONFLICT OF INTEREST STATEMENT

Edward Vitkin, Avshalom Shalom, Julia Wise, Alexander Golberg, and Zohar Yakhini are consultants to Elsy Medical.

## Supporting information


**Fig. S1**. cSCC vs. healthy volcano analysis showing the fold change difference of lipid intensity.

**Fig. S2**. BCC vs. healthy volcano analysis showing the fold change difference of lipid intensity.

**Fig. S3**. cSCC vs. BCC volcano analysis showing the fold change difference of lipid intensity.

**Table S1**. List of all lipids identified.

**Table S2**. cSCC vs. healthy differentially expressed lipids.

**Table S3**. BCC vs. healthy differentially expressed lipids.

**Table S4**. cSCC vs. BCC differentially expressed lipids.

**Supplementary Methods**. More detailed description of UPLC‐MS‐MS analysis

## Data Availability

The data that supports the findings of this study are available in the supplementary material of this article and at https://github.com/GolbergLab/BCC_SCC_Lipidomics.
