# Supplementary figures and images for: High‐throughput lipidomic profiles sampled with electroporation‐based biopsy differentiate healthy skin, cutaneous squamous cell carcinoma, and basal cell carcinoma
Source: Skin Res Technol. 2024 May 9;30(5):e13706. doi: 10.1111/srt.13706 (PMC11079884; doi:10.1111/srt.13706)

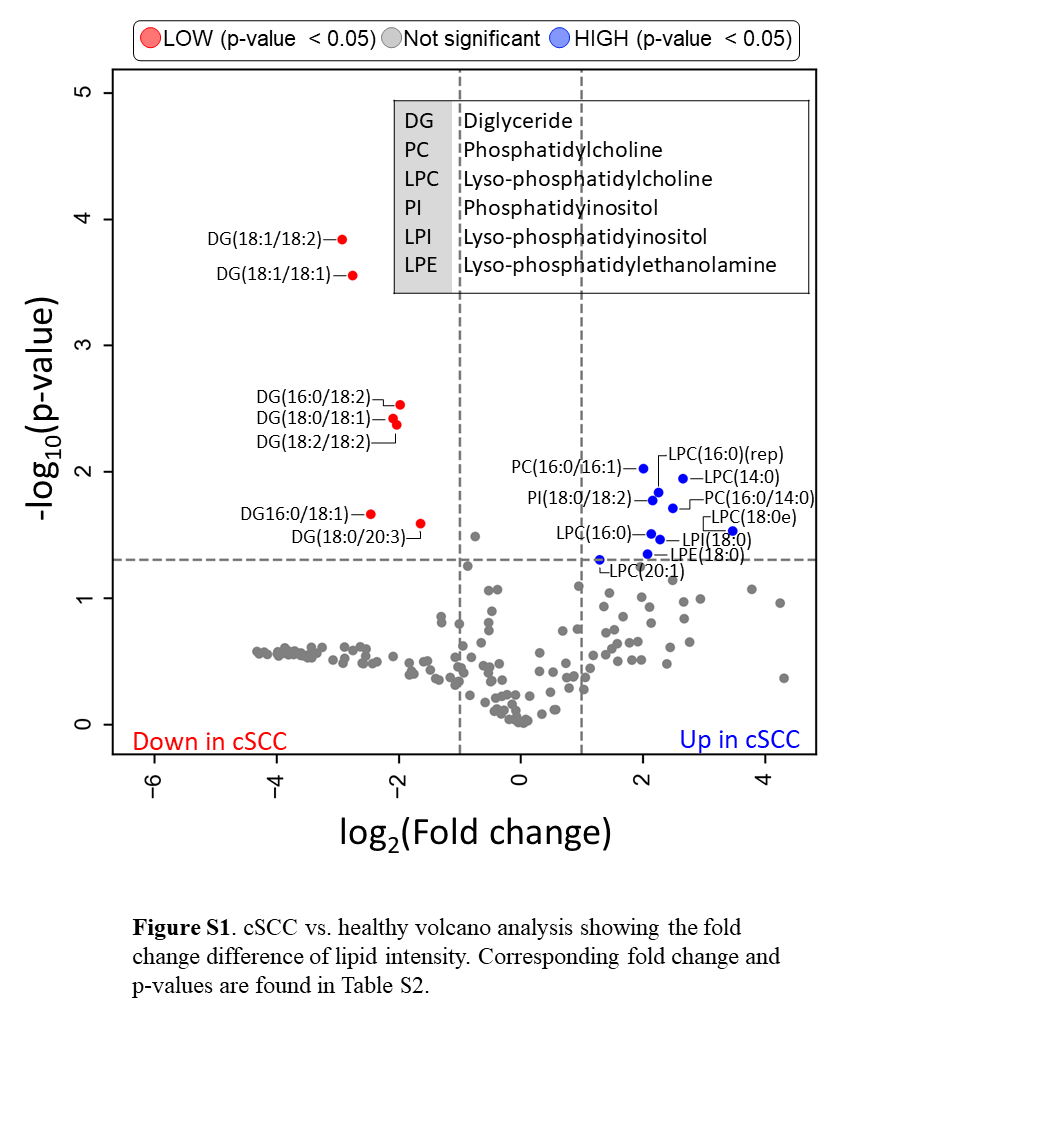

Supplement: Supplementary file 1 — Fig. S1. cSCC vs. healthy volcano analysis showing the fold change difference of lipid intensity. [file SRT-30-e13706-s005.png]

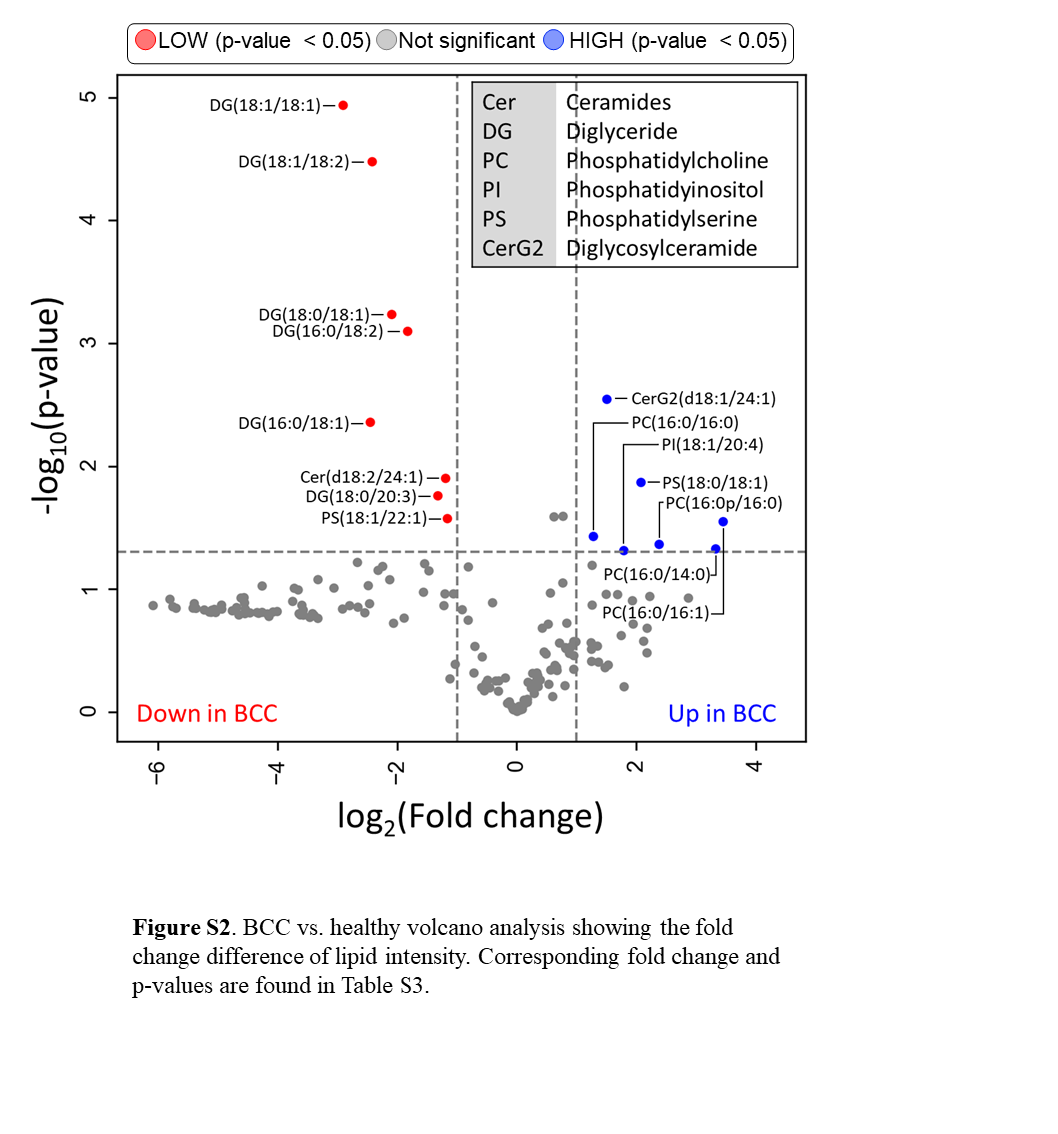

Supplement: Supplementary file 2 — Fig. S2. BCC vs. healthy volcano analysis showing the fold change difference of lipid intensity. [file SRT-30-e13706-s007.png]

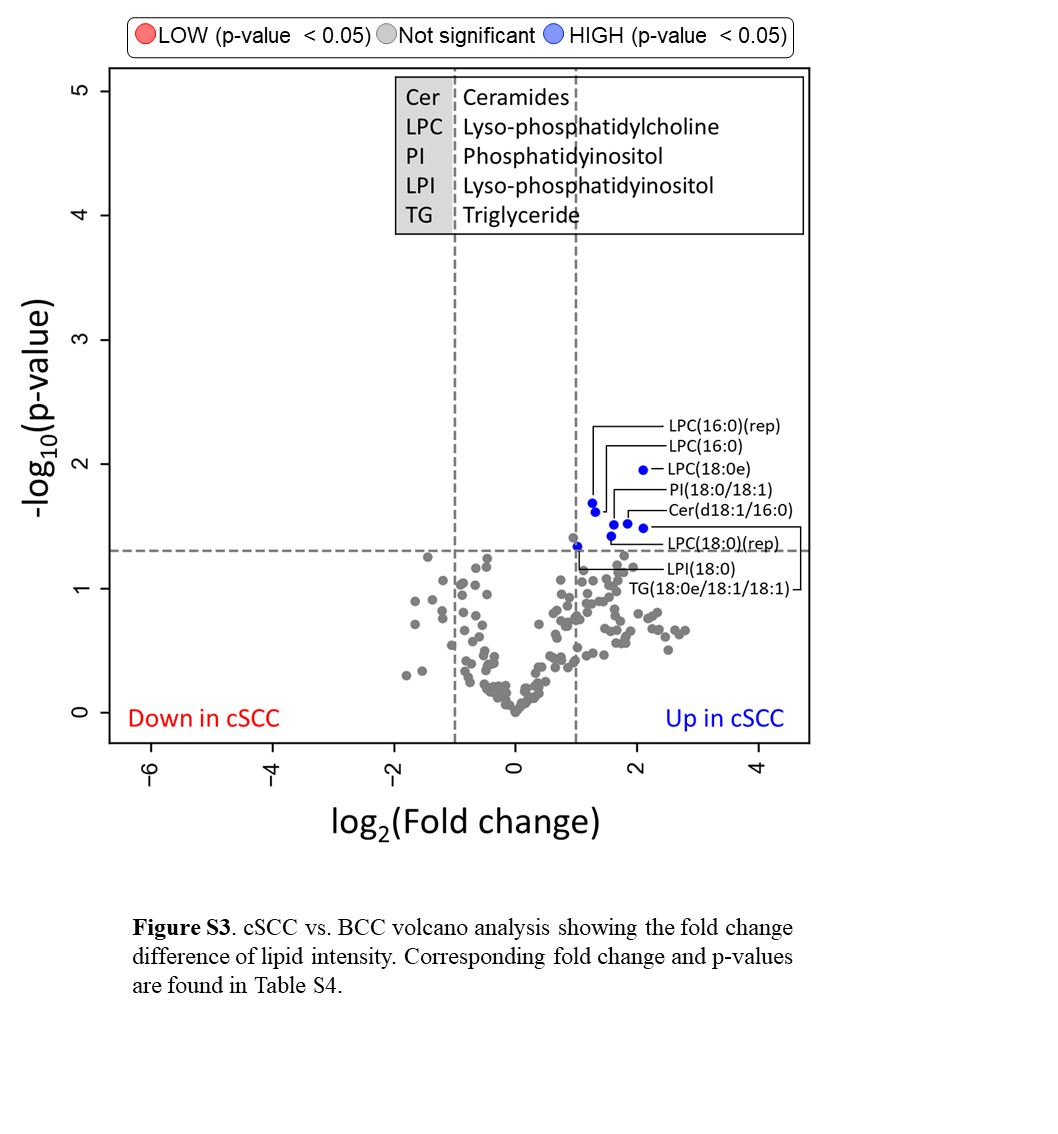

Supplement: Supplementary file 3 — Fig. S3. cSCC vs. BCC volcano analysis showing the fold change difference of lipid intensity. [file SRT-30-e13706-s008.png]
